# Venous thromboembolism in the ICU: main characteristics, diagnosis and thromboprophylaxis

**DOI:** 10.1186/s13054-015-1003-9

**Published:** 2015-08-18

**Authors:** Clémence Minet, Leila Potton, Agnès Bonadona, Rébecca Hamidfar-Roy, Claire Ara Somohano, Maxime Lugosi, Jean-Charles Cartier, Gilbert Ferretti, Carole Schwebel, Jean-François Timsit

**Affiliations:** UJF-Grenoble I, University Hospital Albert Michallon, Medical Intensive Care Unit, Grenoble, F-38041 France; Department of Radiology, UJF-Grenoble I, University Hospital Albert Michallon, Grenoble, F-38041 France; UJF-Grenoble I, University Hospital Albert Michallon, U823 Institut Albert Bonniot, Team 11: Outcome of mechanically ventilated patients and airway cancers, Grenoble, F-38041 France

## Abstract

Venous thromboembolism (VTE), including pulmonary embolism (PE) and deep venous thrombosis (DVT), is a common and severe complication of critical illness. Although well documented in the general population, the prevalence of PE is less known in the ICU, where it is more difficult to diagnose and to treat. Critically ill patients are at high risk of VTE because they combine both general risk factors together with specific ICU risk factors of VTE, like sedation, immobilization, vasopressors or central venous catheter. Compression ultrasonography and computed tomography (CT) scan are the primary tools to diagnose DVT and PE, respectively, in the ICU. CT scan, as well as transesophageal echography, are good for evaluating the severity of PE. Thromboprophylaxis is needed in all ICU patients, mainly with low molecular weight heparin, such as fragmine, which can be used even in cases of non-severe renal failure. Mechanical thromboprophylaxis has to be used if anticoagulation is not possible. Nevertheless, VTE can occur despite well-conducted thromboprophylaxis.

## Introduction

**Fig. 1:**
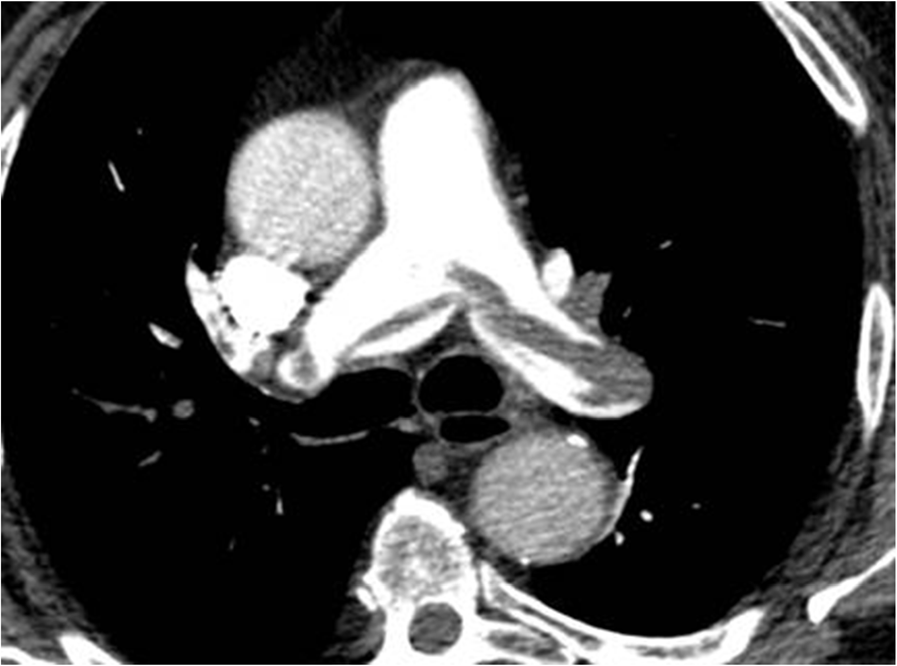
Proximal bilateral pulmonary embolism on computed tomography scan in a mechanically ventilated ICU patient

A 77-year-old man was admitted to the ICU as a result of status epilepticus. He was mechanically ventilated for 3 days, and received 5,000 UI unfractionated heparin (UFH) daily as thromboprophylaxis. The day after his extubation, he became hypoxemic without hypotension. A contrast enhanced computed tomography (CT) scan of the chest (Fig. 1) showed a proximal bilateral pulmonary embolism from the lobar to subsegmental arteries of both sides. Transthoracic echocardiography and CT scan did not show any signs suggestive of right ventricular strain. He recovered with a therapeutic dose of heparin, and was discharged home 1 week later. This clinical case shows that clinical presentation of proximal pulmonary embolism is not typical in mechanical ventilated patients and can occur under thromboprophylaxis.

Venous thromboembolism (VTE), including deep venous thrombosis (DVT) and pulmonary embolism (PE), is a common complication in the ICU. Critically ill patients are at high risk of VTE as they are susceptible to both general risks factors of VTE as well as those specific to ICU patients, such as sedation, immobilization, and vasopressors. The prevalence of VTE, and especially of PE, is underestimated in the ICU, as it is often clinically silent, especially in sedated and mechanically ventilated patients. ICU-acquired thromboembolic events are difficult to diagnose, as they may mimic many other diseases.

Our purpose was to conduct a review of the most relevant published clinical studies on ICU-acquired VTE in order to highlight the main characteristics and the current strategies for the diagnosis and prevention of this disease.

## Search strategy

A search on the PubMed database for English language publications between 1981 and 2014 was performed using search terms “VTE in ICU”, “risk factors of VTE in ICU”, “thromboprophylaxis in ICU”, “pulmonary embolism in ICU”, “deep venous thrombosis in ICU”, “mechanical thromboprophylaxis”, “diagnosis of pulmonary embolism in ICU”.

## Epidemiology

PE is one of the three most frequently underdiagnosed illnesses identified during autopsies [1]. Autopsy studies detected PE in 7 to 27 % of critically ill patients; of these, only one-third were clinically suspected [2].

When PE is clinically suspected, there are three categories of risk: high-risk patients, who are hemodynamically unstable with shock or hypotension; intermediate- to high-risk patients, who are normotensive with a Pulmonary Embolism Severity Index (PESI) ≥ III or a simplified (s)PESI ≥1 and in whom there is right ventricular failure or a rise of cardiac biomarkers; and low risk patients, who have a PESI of class I or II, or a sPESI of 0 [3]. Studies on VTE in the ICU predominantly focus on DVT. The incidence of DVT ranges from 5 to 31 % according to the case-mix and the diagnosis methods used [4–10] (Tables 1 and 2).

**Table 1:** Rate of deep venous thrombosis in critically ill patients without thromboprophylaxis (control groups in randomized clinical trials versus groups with thromboprophylaxis)

Study	Study design	Population	DVT screening method	Number of patients	DVT (%)
Moser et al. 1981 [4]	Prospective cohort	Respiratory ICU	I-labeled fibrinogen leg scanning for 3-6 days	23	13
Cade 1982 [5]	Blinded RCT	General ICU patients	125I-labeled fibrinogen leg scanning for 4-10 days	59	29
Kapoor et al. 1999 [6]	Blinded RCT	Medical ICU patients	CUS at admission and every 3 days	390	31
Fraisse et al. 2000 [7]	Blinded RCT	Exacerbated COPD patients with mechanical ventilation >48 hours	CUS (weekly) and venography (before day 21)	85	28

*COPD* chronic obstructive pulmonary disease; *CUS* compression ultrasonography; *DVT* deep vein thrombosis; *RCT* randomized clinical trial

**Table 2:** Rates of deep vein thrombosis in critically ill patients with thromboprophylaxis

Author (Year)	Study design	Population	DVT screening method	Thromboprophylaxis	Number of patients	DVT (%)
Ibrahim et al. 2002 [10]	Prospective study	Medical ICU patients; MV >7 days	Serial CUS (weekly)	Twice daily SC UFH 5,000 UI	110	23.6
Cook et al. 2005 [9]	Prospective study	Medical-surgical ICU	CUS 48 hours after admission, twice weekly and in case of clinical suspicion	Twice daily SC UFH 5,000 UI	261	9.6
PROTECT 2011 [8]	Blinded RCT	Medical-surgical ICU	CUS 48 hours after admission, twice weekly and in case of clinical suspicion	SC UFH 5,000 UI/dalteparin 5,000 UI plus placebo	3,764	5.4

*CUS* compression ultrasonography; *DVT* deep vein thrombosis; *MV* mechanical ventilation; *RCT* randomized clinical trial; *SC* subcutaneous; *UFH* unfractionated heparin

In a medical-surgical ICU, when compression ultrasonography (CUS) was performed, the incidence of DVT ranged from 10 to 100 % in patients with no clinical suspicion of DVT [11, 12]. Among patients with trauma, routine CT scans discovered asymptomatic PE in 24 % of patients [13].

The prevalence and incidence of PE in the ICU remain unclear. Three recent prospective studies have investigated the incidence of PE in the ICU. A PE was clinically suspected in 0.4 to 2.3 % of medical ICU patients [14, 15]; the incidence rose to 3.2 % in post-traumatic ICU patients [16], although most patients had thromboprophylaxis. In the PROTECT trial comparing prophylaxis with dalteparin versus UFH in critically ill patients (90 % mechanically ventilated), a clinically suspected PE was diagnosed in 1.3 % and 2.3 % of patients, respectively [8].

In 176 consecutive mechanically ventilated patients requiring a CT scan for any medical reason, we applied a standard imaging protocol to detect PE [17]. PE was diagnosed in 33 (18.7 %) patients, and was clinically silent in 20 (60 %) of these [17]. CUS was performed within 48 hours of the CT scan and detected DVT associated with PE in 11 (33 %) patients. Thirty-five (19.9 %) of the 176 patients had a diagnosis of DVT, despite pharmacological thromboprophylaxis or anticoagulant treatment administered to 92 (52.2 %) of them.

PE is associated with a high mortality rate, especially when it is associated with right ventricular failure or shock [18]. Mortality in untreated PE is approximately 30 %, but with adequate (anticoagulant) treatment, this can be reduced to 2 to 8 % [19].

## Diagnosis of ICU-acquired venous thromboembolism

DVT can be ruled out in an outpatient who is judged clinically unlikely to have it and who has a negative D-dimer test [20]. But D-dimer levels can rise in some pathologies like atrial fibrillation, acute coronary syndromes, stroke, acute upper gastrointestinal hemorrhage, infection, disseminated intravascular coagulation, and severe renal dysfunction, which are frequent in critically ill patients. As such, D-dimer levels have low specificity in the ICU [21]. Crowther et al. [22] have shown that neither hypercoagulability nor D-dimer levels predict critically ill patients at risk of DVT and should thus not be used as a diagnostic test for it.

In the general population, CUS is now the first choice to diagnose DVT with high sensitivity and specificity [23]. Outpatients with symptoms of DVT and two consecutively negative CUS exams within 7 days have less than 1 % risk to develop DVT, suggesting that CUS is accurate for ruling out clinically important DVT [24]. DVT can be excluded based on a negative single proximal CUS in patients with low and intermediate clinical probability of DVT [25]. In critically ill patients, an accurate diagnosis of proximal lower extremity DVT can be achieved by intensivists performing lower limb CUS at the bedside, with a sensitivity of 85 % and a specificity of 96 % [26].

A chest CT-based approach has now replaced the gold standard pulmonary angiography with equivalent clinical validity to rule out PE [27]. CT pulmonary angiography (CTPA) has become the imaging reference exam to diagnose PE in the ICU. Multi-detector CT angiography at 1.25 mm collimation thickness is the most sensitive and allows better imaging of segmental and sub-segmental pulmonary arteries [28]. In cases where PE is suspected, multi-detector CT angiography sensitivity ranged between 83 and 100 %, and its specificity ranged between 89 and 97 % [29], although this study did not provide specific data for the ICU population. CT scans can also be useful for determining the severity of acute pulmonary embolisms, by evaluating right ventricular dysfunction, which is well correlated with echocardiography findings [30, 31].

As CTPA requires patients to be transported to the radiology department and in a rather stable state, it cannot be performed in all ICU patients with a clinical suspicion of PE. Moreover, intra-hospital transport may have a negative impact on ventilated critically ill patients [32]. This explains the delayed use of such definitive diagnostic tests in the ICU. Most epidemiologic studies have to be interpreted with caution, as the diagnosis of PE is often based on questionable definitions. We identified only two prospective studies evaluating the rate of PE in critically ill patients with a standardized protocol of CT scans performed in a homogenous consecutive population: one in severe injured trauma patients (24 % PE when scanning asymptomatic severe injured patients) [13]; and one in mechanically ventilated patients (18.7 % PE using a standard imaging protocol to detect PE during CT scan for any other medical reason) [17].

Transthoracic echocardiography (TTE) could be helpful in the diagnosis of PE at the bedside by looking for right ventricular hypokinesis, increases in right ventricular end-diastolic diameter, or tricuspid regurgitation velocity. Miniati et al. [33] showed that TTE could help to assess the physiological effects of PE but that it fails to identify more than 50 % of PE proven on pulmonary angiography. TTE requires better sensitivity for it to be used as a screening test to rule out PE.

Transesophageal echocardiography (TEE) could be useful in PE diagnosis, particularly in hemodynamically unstable patients who have predominantly bilateral central PE. This exam is non-invasive, can be done at the bedside, identifies clots and, compared with CT scanning, has very good sensitivity and specificity (80 % and 100 %, respectively, in Pruszczyk et al.’s study [34]). But TEE has a higher failure rate than CT scans for diagnosis of distal PE.

## Venous thromboembolism risk factors more specific to critically ill patients

ICU patients share similar general risk factors for VTE with other patients: age, immobilization, obesity, past history of personal or familial VTE, past history of neoplasm, sepsis, stroke, respiratory or heart failure, pregnancy, trauma, or recent surgery [9, 17, 35–37].

Additional, specific risk factors for the ICU population have also been described [35, 38] (Table 3).

**Table 3:** Venous thromboembolism risk factors

General VTE risk factors	ICU-acquired VTE risk factor
Age	Sepsis
Past history of VTE	Vasopressor use
Past history of cancer	Respiratory or cardiac failure
Immobilization	Pharmacologic sedation
Obesity	Mechanical ventilation
Pregnancy	Central venous catheter
Trauma, spinal cord injury	End-stage renal failure
Recent surgery	
Stroke	

*VTE* venous thromboembolism

Mechanical ventilation, by decreasing venous return and requiring sedation (and immobilization) increases the risk of VTE. Although critically ill patients with DVT had a longer duration of mechanical ventilation than those who did not [9], the causal relationship between length of mechanical ventilation and VTE is unclear [17]. Sedation is not an independent risk factor in itself.

Central venous catheterization is another important risk factor for ICU-acquired VTE [39], especially when inserted in femoral veins [38, 40], with a catheter-related thrombosis occurrence rate ranging from 2.2 % [8] up to 69 % [17]. Catheter-related thrombosis was originally described by Chastre et al. [41]. The incidence of thrombosis is 2 to 10 % with subclavian catheter [39, 40] but may reach 10 to 69 % with femoral catheter [17, 40, 42, 43] and 40 to 56 % with internal jugular catheter. In superior vena cava catheter-related thrombosis, the risk of associated PE is 7 to 17 % [44, 45]. Lower-limb DVT was associated with a four-fold increase in the risk of PE [17], whereas upper-limb DVT was not a significant risk factor for PE [46–48]. Catheter-related thrombosis risk increases with the duration of catheter placement [10]. In the ICU, catheter-related thrombosis is more frequent in older patients, with femoral catheters, when catheters are inserted in an emergency situation, and in patients not receiving therapeutic heparin [39, 40]. Sepsis may induce procoagulant status and favor catheter-related thrombosis. Catheter-related sepsis is often associated with catheter-related thrombosis [49], and also in ICU patients [39].

Vasopressor administration was found to be an independent risk factor for DVT (hazard ratio 2.8, 95 % confidence interval 1.1 to 7.2) [9], certainly explained by reduced absorption of subcutaneous heparin linked to the vasoconstriction of peripheral blood vessels. This mechanism could explain the lower anti-Xa factor activity after thromboprophylaxis with low molecular weight heparin (LMWH) in critically ill patients on vasopressors [50].

Platelet transfusion (hazard ratio 3.2, 95 % confidence interval 1.2 to 8.4) [9] and high levels of platelets (odds ratio 1.003, 95 % confidence interval 1.000 to 1.006) [17] have been identified as risk factors for VTE, certainly related to increased platelet activation and adherence to vessel walls with subsequent fibrin clot formation, as described in inflammatory processes and sepsis [51].

The level of risk of VTE in critically ill patients also depends on the underlying illness leading to ICU admission [52].

## Is there a rationale to use thromboprophylaxis in critically ill patients?

Thromboprophylaxis is recommended in the general surgical [53] and medical [54] populations. In the ICU, three randomized controlled trials (RCTs), comparing thromboprophylaxis with placebo using objective screening for DVT [5–7] (Table 4), found that the rate of DVT was significantly lower in the thromboprophylaxis group regardless of the thromboprophylaxis used, UFH [5, 6] or LMWH [7]. The American College of Chest Physicians (ACCP) recommends thromboprophylaxis for prevention of VTE in critical care patients (grade Ia: strong recommendation with high quality of evidence) [35, 55]. Moreover, omission of thromboprophylaxis within the first 24 hours of ICU admission without obvious reasons is associated with a higher risk of mortality in the ICU [56].

**Table 4:** Thromboprophylaxis in ICU (blinded randomized controlled trials)

						DVT (%)			PE (%)		
Author (Year)	Population	Number of patients	Diagnosis method	Control	Intervention	Control	Intervention	*P-*value	Control	Intervention	*P-*value
Cade 1982 [5]	General ICU patients	119	125I-labeled fibrinogen leg scanning for 4-10 days	Placebo	UFH 5,000 UI SC twice daily	NR/NR (29 %)	NR/NR (13 %)	<0.05			
Kapoor et al. 1999 [6]	Medical ICU patients	791	CUS at admission and every 3 days	Placebo	UFH 5,000 UI SC twice daily	122/390 (31 %)	44/401 (11 %)	0.001			
Fraisse et al. 2000 [7]	Exacerbated COPD patients with MV >48 hours	223	CUS weekly and venography before day 21	Placebo	Nadroparin 70 UI anti-factor Xa/kg once daily	24/85 (28 %)	13/84 (15.5 %)	<0.045			
PROTECT 2011 [8]	Medico-surgical ICU patients	3764	CUS at admission, twice weekly, and in case of clinical suspicion	5,000 UI SC UFH twice daily	Dalteparin 5,000 UI SC once daily plus placebo	96/1,873 (5.1 %)	109/1,873 (5.8 %)	0.57	43/1,873 (2.3 %)	24/1,873 (1.3 %)	0.01

*COPD* chronic obstructive pulmonary disease; *CUS*, compression ultrasonography; *DVT* deep vein thrombosis; *MV* mechanical ventilation; *NR* not reported; *PE* pulmonary embolism; *SC* subcutaneous; *UFH* unfractionated heparin

In the ICU, up to 80 % of patients under thromboprophylaxis have at least one episode of bleeding, more often minor [57]. Major bleeding is described in 5.6 % of critically ill patients with or without preventive anticoagulation [57], and up to 7.2 % when dalteparin is used in severe renal insufficiency patients [58].

## Which thromboprophylaxis for which patients in the ICU?

### Unfractionated heparin versus low molecular weight heparin

In general surgical patients [59] and medically ill in-patients [60], LMWH and UFH have similar efficacy and safety. In patients with major trauma, enoxaparin (LMWH) was more effective than subcutaneous UFH [61]. In patients with heart failure or severe respiratory disease, enoxaparin 40 mg once daily was as effective as 5,000 UI UFH three times daily for prevention of thromboembolic events [62].

Until now, the PROTECT study [8] is the only RCT to have compared UFH with LMWH as VTE prophylaxis in the ICU, excluding patients at very high risk of bleeding. Overall, 3,764 patients, including 90 % mechanically ventilated patients, were randomly allocated to receive 5,000 UI subcutaneous dalteparin once daily plus placebo once daily, or 5,000 UI subcutaneous UFH twice daily. DVT was screened for using CUS within 48 hours after admission, and then twice weekly or in case of clinical suspicion. There was no significant difference in proximal DVT: 5.1 % DVT in the dalteparin group versus 5.8 % in the UFH group (*P* = 0.57). However, the rate of PE was significantly lower in the dalteparin group (1.3 %) compared with the UFH group (2.3 %) (*P* = 0.01).

A recent review described a significant reduction in PE, but not DVT, with LMWH compared with UFH, with a similar rate of bleeding [63]. ACCP recommends the use of LMWH or UFH thromboprophylaxis in critical care patients at moderate risk for VTE and LMWH for critical care patients at higher risk (major trauma or orthopedic surgery patients) (grade Ia) [35, 55].

### Are low molecular weight heparins easy to use in critically ill patients?

Anti-factor Xa level is a clinically practicable marker of LWMH anticoagulant effectiveness; levels of 0.1 to 0.3 UI/ml are considered as effective antithrombotic activity. There is no need to systematically measure anti-factor Xa levels for each patient but it could be useful in some populations such as critically ill patients because possible patient-dependent factors can influence plasma anti-factor Xa activity, like decreased bioavailability because of edema, vasoconstrictive treatment or renal failure.

Lower anti-factor Xa levels in blood have been reported in ICU patients with generalized edema [64] or receiving vasopressors [50, 65]. This can be explained by impaired peripheral circulation. The systemic bioavailability of the anticoagulant may then be inadequate. However, Priglinger et al. [65] did not show any correlation between dose of norepinephrine and anti-factor Xa blood levels. Robinson et al. [66] conducted a RCT showing that an increase in enoxaparin dose led to significantly increased anti-factor Xa activity but a ceiling effect seems to exist at the dose of 60 mg/day [66]. Mayr et al. also reported an anti-factor Xa activity below the recommended level (0.1 to 0.3 UI/ml) in ICU patients; it was significantly correlated to multiple organ dysfunction as well as to a high body weight [67].

These results suggest that an inadequate dose of enoxaparin can fail to prevent VTE in critically ill patients. Its efficacy should be controlled by monitoring the anti-factor Xa activity in each ICU patient, regardless of the patient’s renal function.

### Low molecular weight heparins and renal insufficiency in critically ill patients

LMWHs are more dependent on renal clearance than UFHs and could bioaccumulate in patients with renal insufficiency, causing more bleeding. Critically ill patients are at higher risk of acute renal failure; at ICU admission, nearly one-third of patients have a creatinine clearance below 30 ml/minute [9]. This reduced renal clearance of LMWH has led to recommendations to monitor LMWH blood levels in patients with severe renal insufficiency [68]. A first meta-analysis failed to demonstrate bioaccumulation of LMWH used as thromboprophylaxis in critically ill patients with renal insufficiency [69]. Two recent prospective observational studies have been performed: the first is a single-center cohort study enrolling 19 patients with a creatinine clearance of 30 ml/minute or above at ICU admission, receiving 5,000 UI subcutaneous dalteparin daily [70]. The second study was a multicenter prospective cohort study of 138 ICU patients with an estimated creatinine clearance under 30 ml/minute [58], and who received 5,000 UI subcutaneous dalteparin once daily for thromboprophylaxis. No bioaccumulation of LMWH occurred in both studies. Dalteparin has not been associated with bleeding in critically ill patients with severe renal insufficiency. However, the impact of LMWHs other than dalteparin is still controversial.

## Mechanical thromboprophylaxis in ICU patients

When anticoagulant is contraindicated, mechanical thromboprophylaxis using either graduated compression stockings (GCS) or intermittent pneumatic compression (IPC) may be proposed. Thromboprophylaxis by mechanical means alone is recommended for critical care patients at high risk of bleeding with contraindications to prophylaxis with anticoagulant agents [35]. The main RCTs that include GCS or IPC for DVT prophylaxis in ICU patients are listed in Table 5 [71]; one was conducted in patients with acute myocardial infarction [72], and three others in trauma patients [73–75]. These four studies represent 791 patients who underwent mechanical prophylaxis (several methods were evaluated) or received LMWH. One study evaluated GCS on one leg versus nothing on the second leg in each patient; the incidence of DVT was lower with GCS (0 % versus 10 %) [72]. Combining IPC with GCS was not more effective than GCS alone [76]. In neurosurgical patients, GCS alone prevented DVT less effectively than when combined with LMWH [77].

**Table 5:** Randomized clinical trials evaluating mechanical thromboprophylaxis in the ICU

Author (year)	Population	Number of patients	Diagnosis method	Intervention	DVT incidence	*P-*value
Kierkegaard and Norgren 1993 [72]	Patients aged >70 years, myocardial infarction	80	I-labeled fibrinogen test on alternate days	No GCS	8 (10 %)	0.003
				GCS	0 (0 %)	
Elliott et al. 1999 [74]	Trauma patients	149	CUS on day 8 or before	Calf-thigh IPC	4 (6.5 %)	0.009
				Plantar venous IPC	13 (21 %)	
Ginzburg et al. 2003 [75]	Trauma patients	442	CUS within 24 hours of admission and weekly	IPC	6 (2.7 %)	0.12
				LMWH	1 (0.5 %)	
Kurtoglu et al. 2004 [73]	Trauma patients	120	CUS on admission, weekly, and 1 week after discharge	IPC	4 (6.6 %)	0.04
				LMWH	3 (5 %)	

*CUS* compression ultrasonography; *DVT* deep vein thrombosis; *GCS* graduated compression stockings; *IPC* intermittent pneumatic compression; *LMWH* low molecular weight heparin

The use of vena cava filters for thromboprophylaxis is not recommended by the ACCP Evidence-Based Clinical Practice Guidelines (eighth edition) [35].

### Thromboprophylaxis compliance in the ICU

The ENDORSE multinational study enrolled 68,183 hospitalized patients in an acute care setting and showed that only a low rate of patients had appropriate prophylaxis according to the 2004 ACCP guidelines on VTE prophylaxis. In an Asian ICU, a recent observational study revealed that 20 % of the critically ill patients did not receive the appropriate recommended prophylaxis [78]. In North-American ICUs, Lauzier et al. [79] recently reported appropriate guideline concordance occurred for 95.5 % patient-days, which was better in sicker patients and in patients with a previous history of VTE or cancer. LMWH was less used than UHF in sicker and surgical patients, and in patients receiving vasopressors or renal replacement therapy [79].

## What about asymptomatic pulmonary embolism?

The prevalence of incidental emboli in in-patients varies from 0.6 to 5.7 % [80]. In moderate to severe injured trauma patients, CT scans showed asymptomatic PE in 22 (24 %) patients, of which 30 % were receiving thromboprophylaxis. Only four patients with a major clot, and one with a minor clot but with an associated DVT, were treated at therapeutic doses. None of the 10 patients with minor clot and no therapeutic anticoagulation therapy was associated with any complication attributable to thromboembolic disease within 3 months [13]. In our study of mechanically ventilated patients in the ICU, 33 of 176 (18.7 %) patients had a diagnosed PE; 20 of these were asymptomatic (60 %) [17]. All these patients were treated early with therapeutic anticoagulation whenever they had lobar, segmental or subsegmental PE. ICU and hospital mortality were not different between patients with and without diagnosed PE.

So what should we do for asymptomatic PE cases? Should we routinely look to detect all asymptomatic PE? Although it seems evident to treat all PE, regardless of their symptoms, once they are detected, should we treat all asymptomatic PE cases? Most unsuspected emboli found on CT scans are small and segmental or subsegmental [81] and their management is not clear. Patients with false negative undiagnosed PE on CT scan have favorable short-term outcome without therapeutic anticoagulation [82]. Eyer et al. [83] also demonstrated no attributable mortality with untreated subsegmental emboli. On the other hand, early diagnosis of PE and routine use of early therapeutic anticoagulation in symptomatic or asymptomatic PE contributed to a low rate of fatal PE [17]. These results seem to support the need for routinely looking for PE in patients receiving mechanical ventilation. Whether mortality would be higher if these asymptomatic PE cases are not treated remains to be evaluated.

## Conclusion

Diagnosis and management of clinically silent PE in critically ill patients are challenging. Current diagnosis tools, such as CTPA, allow the efficient diagnosis of silent PE if carried out systematically in mechanically ventilated patients. Considering the high risk of VTE in ICU patients, including specific VTE risk factors like mechanical ventilation, vasopressor use and central venous catheter use, thromboprophylaxis is recommended. LMWH could be more effective than UFH for VTE prophylaxis in the ICU. However, the high risk of bleeding in many critically ill patients makes the benefit-risk ratio of thromboprophylaxis difficult to evaluate. Further research on the diagnosis of PE and on whether asymptomatic peripheral PE should be treated or not is needed.

## Abbreviations

ACCP: American College of Chest Physicians
CT: computed tomography
CTPA: computed tomographic pulmonary angiography
CUS: compression ultrasonography
DVT: deep venous thrombosis
GCS: graduated compression stockings
IPC: intermittent pneumatic compression
LMWH: low molecular weight heparin
PE: pulmonary embolism
PESI: Pulmonary Embolism Severity Index
RCT: randomized controlled trial
sPESI: simplified Pulmonary Embolism Severity Index
TEE: transesophageal echocardiography
TTE: transthoracic echocardiography
UFH: unfractionated heparin
VTE: venous thromboembolism
